# Supplementary material for: Comparative genomics provides new insights into the diversity, physiology, and sexuality of the only industrially exploited tremellomycete: Phaffia rhodozyma
Source: BMC Genomics. 2016 Nov 9;17:901. doi: 10.1186/s12864-016-3244-7 (PMC5103461; doi:10.1186/s12864-016-3244-7)
Supplement: Additional file 6: — List of orphan genes with links to PFAM (related to Additional file 1: Table S1). (ZIP 1428 kb) [file 12864_2016_3244_MOESM6_ESM.zip › PFAM_UNIQUE/G02824_P.html]

#                                                                            --- full sequence --- -------------- this domain -------------   hmm coord   ali coord   env coord
  
# target name        accession   tlen query name           accession   qlen   E-value  score  bias   #  of  c-Evalue  i-Evalue  score  bias  from    to  from    to  from    to  acc description of target
  
#------------------- ---------- ----- -------------------- ---------- ----- --------- ------ ----- --- --- --------- --------- ------ ----- ----- ----- ----- ----- ----- ----- ---- ---------------------
  
G02824\_P             -            377 Adaptin\_binding      PF10199.4    137       7.3    5.5   9.9   1   2      0.37   1.1e+02    1.8   0.1    48    85    45    90    36   132 0.65 - Alpha and gamma adaptin binding protein p34  
G02824\_P             -            377 Adaptin\_binding      PF10199.4    137       7.3    5.5   9.9   2   2     0.047        13    4.7   6.5     3    65   288   346   286   352 0.74 - Alpha and gamma adaptin binding protein p34  
G02824\_P             -            377 CDC45                PF02724.9    622      0.82    6.3   8.8   1   2      0.17        16    2.0   0.0   119   149    50    81    31   177 0.65 - CDC45-like protein  
G02824\_P             -            377 CDC45                PF02724.9    622      0.82    6.3   8.8   2   2     0.024       2.2    4.9   6.2   133   169   308   343   270   371 0.52 - CDC45-like protein  
G02824\_P             -            377 DUF2052              PF09747.4    181       2.3    6.8  12.4   1   2      0.15        41    2.7   0.6    60    98    38    79     9   108 0.53 - Coiled-coil domain containing protein (DUF2052)  
G02824\_P             -            377 DUF2052              PF09747.4    181       2.3    6.8  12.4   2   2    0.0029       0.8    8.3   5.3    55   108   296   344   271   374 0.46 - Coiled-coil domain containing protein (DUF2052)  
G02824\_P             -            377 MCPVI                PF02993.9    238      0.73    8.6   7.0   1   2   2.2e-05     0.044   12.6   0.5   114   227   179   300   149   305 0.65 - Minor capsid protein VI  
G02824\_P             -            377 MCPVI                PF02993.9    238      0.73    8.6   7.0   2   2      0.44   8.8e+02   -1.5   1.2   105   154   317   366   309   375 0.49 - Minor capsid protein VI  
G02824\_P             -            377 Na\_trans\_assoc       PF06512.8    239      0.23   10.0   5.6   1   2    0.0078       6.7    5.3   0.0    32    95    42   102    30   111 0.57 - Sodium ion transport-associated  
G02824\_P             -            377 Na\_trans\_assoc       PF06512.8    239      0.23   10.0   5.6   2   2     0.016        14    4.2   4.0   155   187   312   339   239   350 0.66 - Sodium ion transport-associated  
G02824\_P             -            377 PBP1\_TM              PF14812.1     81      0.53    9.3  21.6   1   2    0.0016      0.25   10.4   6.9    15    51    43    81    36   100 0.61 - Transmembrane domain of transglycosylase PBP1 at N-terminal  
G02824\_P             -            377 PBP1\_TM              PF14812.1     81      0.53    9.3  21.6   2   2      0.12        19    4.3   6.7    32    59   311   339   276   353 0.74 - Transmembrane domain of transglycosylase PBP1 at N-terminal  
G02824\_P             -            377 PBP\_sp32             PF07222.7    243      0.04   11.8   2.9   1   1   7.9e-05     0.079   10.8   2.9   215   243   315   343   237   351 0.85 - Proacrosin binding protein sp32  
G02824\_P             -            377 Prothymosin          PF03247.9    106       1.1    8.2  31.8   1   2       2.9   3.9e+02   -0.1   8.9    53    91    58    97    43   105 0.43 - Prothymosin/parathymosin family  
G02824\_P             -            377 Prothymosin          PF03247.9    106       1.1    8.2  31.8   2   2   0.00014     0.018   13.8  14.9    17    79   277   346   268   356 0.53 - Prothymosin/parathymosin family  
G02824\_P             -            377 RNA\_pol\_3\_Rpc31      PF11705.3    233      0.88    8.1  18.0   1   2     0.046        14    4.1   0.8   174   203    52    80    16    83 0.63 - DNA-directed RNA polymerase III subunit Rpc31  
G02824\_P             -            377 RNA\_pol\_3\_Rpc31      PF11705.3    233      0.88    8.1  18.0   2   2    0.0023      0.72    8.4  10.4   154   210   287   344   255   347 0.70 - DNA-directed RNA polymerase III subunit Rpc31  
G02824\_P             -            377 SDA1                 PF05285.7    324       6.5    4.6  24.5   1   2      0.34        35    2.2   5.7    83   133    48    99    38   110 0.53 - SDA1  
G02824\_P             -            377 SDA1                 PF05285.7    324       6.5    4.6  24.5   2   2    0.0049      0.49    8.3  10.9   101   143   290   346   271   375 0.53 - SDA1  
G02824\_P             -            377 Spore\_coat\_CotO      PF14153.1    185     0.017   13.2   5.1   1   2      0.05        11    4.1   0.0    42    98    44   100     4   167 0.78 - Spore coat protein CotO  
G02824\_P             -            377 Spore\_coat\_CotO      PF14153.1    185     0.017   13.2   5.1   2   2    0.0033      0.71    7.9   4.5    74   112   308   346   271   375 0.51 - Spore coat protein CotO  
G02824\_P             -            377 TALPID3              PF15324.1   1252         4    3.3   7.6   1   1    0.0054       5.4    2.9   7.6   930  1032   182   290   152   348 0.70 - Hedgehog signalling target
